# Comprehensive analysis of vaginal microbiota, metabolites, and inflammatory factors in preterm and term pregnancies

**DOI:** 10.3389/fmicb.2025.1689494

**Published:** 2025-10-02

**Authors:** Li-Ping Shen, Bei-Jun Cai, Jing-Xue Guan, Ting Peng, Lei Jin

**Affiliations:** ^1^Department of Obstetrics, Changning Maternity and Infant Health Hospital, East China Normal University, Shanghai, China; ^2^Department of Rheumatology, Immunology & Allergy, Shanghai General Hospital, Shanghai Jiao Tong University School of Medicine, Shanghai, China

**Keywords:** preterm birth, vaginal microbiota, *Lactobacillus jensenii*, metabolites, SII

## Abstract

**Purpose:**

This study aimed to reveal the interrelationships among vaginal microorganisms, metabolism, and inflammatory factors in premature pregnant women.

**Methods:**

A total of 77 pregnant women were enrolled and divided into a preterm birth group (*n* = 23) and a full-term birth group (*n* = 54) according to the gestational week of delivery. Blood samples and vaginal secretion samples were collected before the onset of labor or after rupture of membranes for blood index testing, 16S RNA sequencing of vaginal secretion samples, and untargeted metabolite determination.

**Results:**

Compared with the full-term group, the preterm group exhibited significantly elevated inflammatory markers (SII 689 vs. 1,061, *p* < 0.001) and decreased vaginal microbiota *α* diversity (Shannon index 3.56 vs. 2.65). Meanwhile, the abundance of *Firmicutes* was increased (54.96% vs. 76.73%), primarily comprising *Lactobacillus jensenii*, which was negatively correlated with gestational week; metabolomics identified 83 significantly differential metabolites, including upregulated tyrosine-arginine, cholesterol sulfate, and benzene compounds such as 2,4-dichlorophenol. KEGG analysis revealed that pathways such as kynurenine, steroids, lipids, and microbial metabolism were significantly activated in the preterm birth group. Omics association analysis revealed significant correlations among microbiota, metabolites, and inflammatory markers. For example, *Lactobacillus jensenii* and inflammatory metabolites such as arginine-lysine, sulfamethoxazole, 5-aminovaleric acid, and epoxiconazole were all positively correlated with SII (*p* < 0.05).

**Conclusion:**

The results suggest that an imbalance in vaginal microbiota, particularly the abnormal proliferation of *Lactobacillus jensenii,* as well as amino acid and lipid metabolism may be associated with inflammation-induced preterm birth.

## 1 Introduction

Spontaneous preterm birth (sPTB) is one of the leading causes of neonatal mortality and long-term complications worldwide. There are approximately 15 million cases of preterm birth each year, resulting in enormous healthcare burdens and socioeconomic pressure (GBD 2021 Diseases and Injuries Collaborators, 2024; Lipp et al., 2025; Marret et al., 2007). The occurrence of sPTB is associated with pregnancy risk factors, such as short cervix, region, maternal age (younger than 25 or older than 35 years), body mass index (BMI < 18 or BMI > 28), low socioeconomic status, smoking, and genetic polymorphisms (Adane et al., 2024); these risk factors are usually closely related to the maternal physiological and pathological status, and early identification and intervention are crucial for improving pregnancy outcomes (Kindinger et al., 2016). Currently, the prediction of preterm birth primarily relies on clinical history and ultrasound examinations, but there is a lack of reliable biomarkers. Therefore, exploring characteristic biological markers to identify preterm birth could help reduce the risk of preterm birth and its related complications, holding significant clinical implications.

The vaginal microbiome is a dynamic ecological balance system composed of the vaginal microbiota, the host’s endocrine system, vaginal anatomical structure, and local mucosal immunity (Fettweis et al., 2019; Jiang et al., 2025). A normal vaginal microbiome has important defensive functions and plays an irreplaceable role in resisting the invasion of pathogenic microorganisms. The microbiota dominated by *Lactobacillus* as long been considered a sign of female reproductive health (Łaniewski et al., 2020). In recent years, studies have found that an association between the dynamic changes in vaginal microbiota and their metabolites in pregnant women and preterm birth, and the metabolic characteristics of vaginal microbiota can alter in response to changes in the microbiota itself. Previous studies have shown that vaginal microbiota dysbiosis (such as a reduction in *Lactobacillus*) relates to an increased risk of preterm birth (Kindschuh et al., 2023). Metabolites produced by microorganisms can enhance the synthesis levels of local or systemic inflammatory cytokines and interstitial collagenases, thereby inducing preterm birth (Menon, 2022). Several metabolic small molecules produced by *Lactobacillus* are associated with antimicrobial and anti-inflammatory functions, revealing a direct correlation with the risk of preterm birth (Anton et al., 2018). Furthermore, recent studies have found that vaginal metabolites have the potential to serve as early biomarkers for sPTB, and highlighted exogenous exposure as a potential risk factor for preterm birth (Kindschuh et al., 2023). It can be seen that the interaction pattern between microorganisms and metabolic small molecules remains a hot topic of research. The vaginal microbiota may influence pregnancy outcomes by regulating the local immune and metabolic microenvironment, suggesting its potential as a predictive target for preterm birth (Zhang et al., 2025a). These findings provide scientific evidence for uncovering the mechanisms of preterm birth and developing new intervention strategies.

This study employed 16S rRNA sequencing and LC–MS metabolomics technology, combined with clinical inflammation data, to conduct multi-omics analysis, identify vaginal microbiota and characteristic metabolites associated with preterm birth, and explore their underlying mechanisms in relation to inflammation markers. Through a retrospective analysis and rigorous clinical phenotype matching, our research will provide new insights for the prediction and intervention of preterm birth.

## 2 Materials and methods

### 2.1 Subject recruitment and sample collection

All participants were pregnant women who underwent prenatal examination in our hospital from March 2023 to March 2024. Subjects were recruited in accordance with the inclusion criteria (signing the informed consent for the project, with priority given to pregnant women with a history of preterm birth) and exclusion criteria (unwillingness to sign the informed consent for the project, preterm birth due to cervical insufficiency, iatrogenic preterm birth, pregnant women with twin or multiple pregnancies, and those complicated with malignant tumors). Clinical basic information, medical history, pregnancy outcomes, and laboratory test results were collected using the hospital medical history system. Prior to the onset of labour (2–3 regular uterine contractions within 10 min) or after spontaneous rupture of membranes, a vaginal examination was performed using a disposable speculum. Two cotton swabs were dipped in an appropriate amount of secretions from the posterior vaginal fornix and then placed into a sterile sampling tube, with a unique identification code labeled. All samples were stored in a − 80 °C freezer until further use.

### 2.2 Clinical data collection

Collect and record the participants’ clinical basic information, pregnancy outcomes, and pre-delivery blood indices. Clinical baseline information includes maternal age, weight gain (kg), gravidity, parity, smoking history, and assisted reproductive technology status; pregnancy outcomes included gestational age at delivery; blood indices included: white blood cell (WBC), neutrophil count (NEU), lymphocyte count, monocyte% (MONO), platelet count (PLT), basophil% (BASO), eosinophil% (EO), red blood cell (RBC), hemoglobin (HB), systemic immune-inflammation index (SII), total cholesterol (TCHO), low-density lipoprotein (LDL), high-density lipoprotein (HDL), triglyceride (TG), serum lipoprotein a (LPa), apolipoprotein AI (APOAI), and apolipoprotein B (APOB). Based on the gestational age at delivery for all participants, they were assigned to two groups: spontaneous preterm birth group (sPTB) and full-term birth group (FTB).

### 2.3 Vaginal microbiota sequencing

The vaginal microbiota was sequenced and analysed using the Omicsmart platform, following the methodology described in the previous report. Briefly, total microbial DNA was extracted from vaginal secretion samples using a commercially available DNA extraction kit (D3141, Guangzhou Meiji Biotechnology Co., Ltd., China). Subsequently, broad-spectrum bacterial primers, namely primers: according to the selected sequencing region, the V3-V4 region of the bacterial 16S rRNA gene was amplified with the 341F primer (5’-CCTACGGGNGGCWGCAG-3′) and the 806R primer (5’-GGACTACHVGGGTATCTAAT-3′). These products were purified by 2.0% agarose gel electrophoresis, and the target fragments were collected and recovered using the Agencourt Ampure XP kit (Beckman Coulter, Inc., United States). DNA quantitative detection was performed on 77 samples using the ABI StepOnePlus Real-Time PCR System (Life Technologies, United States). Sequencing libraries were constructed and sequenced on the Illumina PE250 platform.

### 2.4 Untargeted metabolomics chromatography detection

Vaginal contents were freeze-dried, weighted, and extracted using an organic solution (methanol: acetonitrile: water = 1:1:1). The solution was thoroughly mixed by vibration using a high-throughput oscillator and placed in an environment of 0–4 °C. After standing for 2 h, the supernatant of each sample was collected by centrifugation (14,000 rpm, 20 min, 4 °C), and then dried at 25 °C under a vacuum environment. The residue was reconstituted with 100 μL of organic solution (acetonitrile: water = 1:1), followed by centrifugation (14,000 rpm, 15 min, 4 °C), and then the supernatant was filtered through a 0.22 μm water membrane. Quality control samples contain equal volumes of each sample, which are used to detect and evaluate the stability of the system and the reliability of experimental data.

### 2.5 Statistical analysis

Statistical analysis of the data was conducted using R software version 4.3.2. Normality of distribution was assessed using the Shapiro–Wilk test. Normally distributed data were expressed as (x ± SD), and comparison between groups of normally distributed measurement data were performed using the two independent samples t-test. For non-normally distributed continuous variables, median (M) and percentiles (P25, P75) were used. Intergroup comparisons of skewed continuous data were conducted using the Mann–Whitney U test. Categorical data were analysed using the chi-square test or Fisher’s exact probability test.

### 2.6 Vaginal microbiota sequences

After sequencing, data filtering, sequence splicing and tag filtering were performed on the raw data. Then, OTU clustering and chimera removal were performed using Usearch (Edgar, 2010) (version 11.0.667). High-quality sequences were collected, and operational taxonomic units (OTUs) with ≥97% similarity were grouped accordingly. The OTU sequences were aligned with the SILVA and NCBI databases (Pruesse et al., 2007), and species classification annotation was completed using the RDP annotation software (Wang et al., 2007). Species-related analyses were performed using the psych package. Indicator species abundance was plotted and analysed using R language. Vegan diversity (*α*/*β* diversity) of the vaginal microbiota was calculated using the R language Vegan package. Based on Bray-Curtis distance, the R software Vegan package was utilised to conduct principal component analysis (PCA), principal coordinate analysis (PCoA) and non-metric multidimensional scaling (NMDS) on the vaginal microbiota of all samples. Bacterial microbial phenotypes were classified using BugBase (version 1.0). Pearson correlation coefficients between pregnant women’s clinical data and species were calculated using the psych package in R language.

### 2.7 Untargeted metabolomics analysis

Vaginal metabolic profiling was performed using a Vanquish LC ultra-high performance liquid chromatography (UHPLC) system with a HILIC column. MS detection of metabolites was carried out on an Orbitrap ExplorisTM 480 (Thermo Fisher, United States) with an ESI ion source operating in positive and negative modes. Mobile phase A contained water + 25 mm ammonium acetate + 25 mm ammonia, and mobile phase B was acetonitrile. Raw data were subjected to peak identification, peak filtering, peak alignment, and normalisation. Based on high-resolution mass spectrometry (HRMS) detection technology, the untargeted metabolome aimed to identify as many molecular feature peaks in the samples as possible. Substance annotation was performed by matching with public databases such as Mass Bank, Metlin, and MoNA, combined with a self-built secondary mass spectrometry database. Principal component analysis (PCA) was conducted on vaginal metabolomics using R language gmodels (v2.18.1). Partial least squares discriminant analysis (PLS-DA, cross-validation: 5-fold) and orthogonal partial least squares discriminant analysis (OPLS-DA, permutation number: 20) were calculated using R language ropls package. Differential metabolic features between the TB group and PTB group [VIP > 1.0, *p* < 0.05, FC > 1] were processed by logarithmic transformation (log10) and auto-scaling (centered on the mean and divided by the standard deviation of each variable), and then visualized by R software.

## 3 Results

### 3.1 Clinical date characteristics of preterm pregnant women

This study included a total of 77 pregnant women who underwent prenatal check-ups and delivered at the Changning District Maternal and Child Health Care Hospital in Shanghai from March 2023 to March 2024, including 54 in the full-term birth group (FTB) and 23 in the spontaneous preterm birth group (sPTB). The clinical characteristics of the enrolled pregnant women are presented in Table 1. The gestational age of the sPTB group was significantly lower than that of the FTB group (*p* < 0.001), the weight gain during pregnancy was significantly less in the sPTB group compared to the FTB group (*p* < 0.001), and the number of cases of membrane rupture was significantly higher in the sPTB group than in the FTB group (*p* = 0.005, < 0.05); however, there were no significant differences in maternal age, gravidity, parity, smoking, and assisted reproduction (*p* > 0.05). Similarly, in the sPTB group, the serum white blood cell count (WBC) before the onset of labor was significantly higher than that in the FTB group (*p* = 0.024, <0.05), the lymphocyte percentage (LYM%) was significantly lower than that in the FTB group (*p* = 0.019, <0.05), the eosinophil percentage (EO%) was significantly lower than that in the FTB group (*p* = 0.02, <0.05), the neutrophil count (NEU) was significantly higher than that in the FTB group (*p* = 0.017, <0.05), the platelet count (PLT) was significantly higher than that in the FTB group (*p* = 0.013, <0.05), and the systemic immune-inflammation index (SII) was significantly higher than that in the FTB group (*p* < 0.001); while there were no significant differences in monocyte percentage (MONO%), basophil percentage (BASO%), red blood cell count (RBC), hemoglobin (HGB), and various blood lipid indexes (*p* > 0.05).

**Table 1:** Clinical data characteristics of sPTB and FTB groups.

Characteristics	FTB	sPTB	*P*
	(*n* = 54)	(*n* = 23)	
Age	30.74 ± 3.38	31.13 ± 3.97	0.662
Gestational age (weeks)	39.00 (38.00, 39.00)	35.00 (34.00, 36.00)	<0.001
Weight gain during pregnancy(kg)	12.50 (10.62, 15.00)	8.50 (7.00, 11.00)	<0.001
Membrane rupture			0.005
No	33 (61.11%)	6 (26.09%)	
Yes	21 (38.89%)	17 (73.91%)	
Gravidity			0.323
1	31 (57.41%)	18 (78.26%)	
2	15 (27.78%)	2 (8.70%)	
3	6 (11.11%)	2 (8.70%)	
4	1 (1.85%)	1 (4.35%)	
5	1 (1.85%)	0 (0.00%)	
Parity			0.207
1	39 (72.22%)	20 (86.96%)	
2	14 (25.93%)	2 (8.70%)	
3	1 (1.85%)	1 (4.35%)	
Smoking			0.529
No	53 (98.15%)	22 (95.65%)	
Yes	1 (1.85%)	1 (4.35%)	
Assisted reproduction			0.529
No	53 (98.15%)	22 (95.65%)	
Yes	1 (1.85%)	1 (4.35%)	
WBC (×10^9/L)	8.47 (7.66,9.03)	9.80 (7.37,12.28)	0.024
MONO%	7.44 ± 1.66	7.03 ± 1.80	0.33
LYM%	20.90 ± 5.48	17.32 ± 7.20	0.019
BASO%	0.20 (0.20,0.30)	0.20 (0.15,0.30)	0.657
EO%	0.70 (0.42,1.17)	0.40 (0.20,0.75)	0.02
NEU	5.80 (5.12,6.88)	7.00 (5.55,9.40)	0.017
PLT (×10^9/L)	189.63 ± 47.03	218.04 ± 39.26	0.013
RBC (×10^12/L)	3.92 (3.71,4.09)	3.82 (3.61,4.04)	0.268
HGB (g/L)	121.00 (116.00,128.00)	117.00 (108.00,126.50)	0.191
LDL (mmol/L)	3.49 ± 0.90	3.83 ± 1.08	0.159
TG (mmol/L)	3.00 (2.37,3.79)	2.91 (2.33,3.54)	0.726
HDL (mmol/L)	1.86 (1.72,2.13)	1.96 (1.69,2.10)	0.872
LPa (mg/dL)	8.20 (3.9,20.57)	19.00 (3.75,47.75)	0.273
APOAI (mg/dL)	220.70 ± 33.66	220.48 ± 35.38	0.979
APOB (mg/dL)	127.85 ± 25.49	136.91 ± 28.78	0.174
TCHO	6.43 ± 1.02	6.70 ± 1.23	0.311
SII	689.00 (465.50,869.75)	1061.00 (634.50,1306.00)	<0.001

WBC (White Blood Cell Count), MONO% (Monocyte Percentage), LYM% (Lymphocyte Percentage), BASO% (Basophil Percentage), EO% (Eosinophil Percentage), NEU (Neutrophil Count), PLT (Platelet Count), RBC (Red Blood Cell Count), HGB (Hemoglobin), LDL (Low-Density Lipoprotein), TG (Triglyceride), HDL (High-Density Lipoprotein), LPa (Serum Lipoprotein a), APOAI (Apolipoprotein AI), APOB (Apolipoprotein B), TCHO (Total Cholesterol), SII (Systemic Immune-Inflammation Index).

### 3.2 Changes in vaginal microbiota diversity in preterm pregnant women

**Figure 1:**
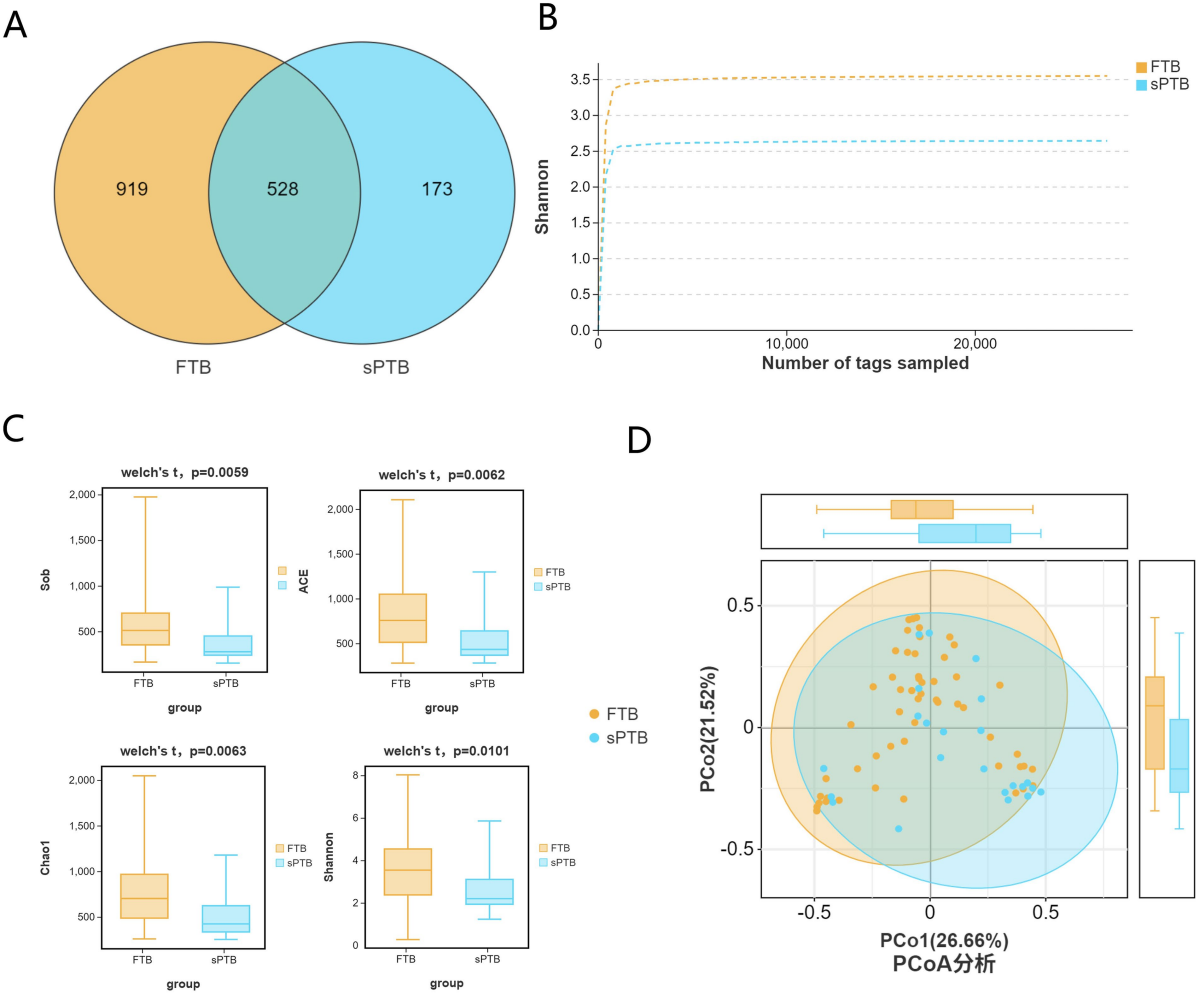
Diversity of Vaginal microbiota in pregnant women across two groups: **(A)** Venn diagram was constructed to illustrate the shared and unique amplicon sequence variants between the FTP and sPTB groups. **(B)** Rarefaction curves were generated for the FTP and sPTB groups to evaluate the sampling depth and coverage of microbial diversity. **(C)** *α* diversity analysis was performed using the Sob/ACE/Chao 1/Shannon indices to compare microbial diversity between the FTP and sPTB groups. **(D)** *β* diversity analysis using PCoA revealed similarities in microbial composition between the FTP and sPTB groups.

Venn diagrams were used to represent the shared and unique amplicon sequences between the FTP and sPTB groups; the rarefaction curve was used to assess the complexity and evenness of *α* diversity in the vaginal microbiota of the two groups; Sob, ACE, Chao1, and Shannon indices are important parameters for assessing species diversity in α diversity analysis; PCoA can be employed to reveal *β* diversity analysis between the term Control group and the premature labor group to reveal the differences in vaginal microbiota composition. As shown in Figure 1, the Venn diagram indicates that the FTB group and sPTB group had 919 and 173 unique OTU sequences, respectively, and 528 shared sequences (Figure 1A). The rarefaction curve indicated that the vaginal microbiota of the FTB group had greater complexity and evenness compared to the sPTB group (Figure 1B). According to Welch’s statistical test, the Sob (383.87), ACE (574.04), Chao1 (545.19), and Shannon (2.65) indices were significantly lower than those in the FTB group (583.22, 817.9, 772.01, and 3.56, respectively) (*p* < 0.05), indicating that changes in vaginal microbiota *α* diversity may serve as an important parameter for predicting preterm birth risk (Figure 1C). The PCoA score plot represented vaginal microbiota *β*-diversity. When the first principal coordinate accounted for 26.66% and the second principal coordinate accounted for 21.52%, the vaginal microbiota composition between the FTB and sPTB groups showed obvious clustering (Figure 1D).

### 3.3 Differences in the taxonomic and functional characteristics of the vaginal microbiota in preterm pregnant women

**Figure 2:**
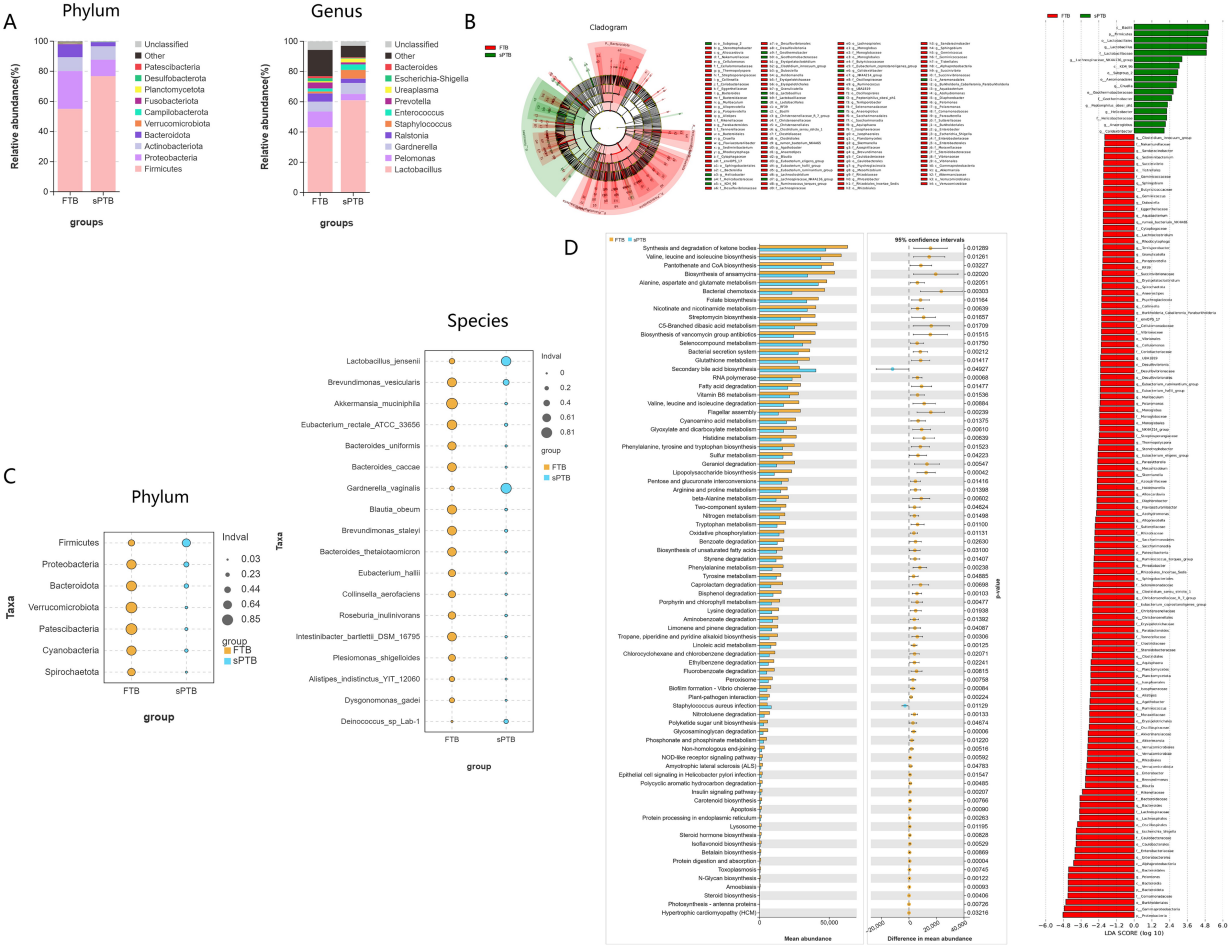
Composition of vaginal microbiota, key microbial communities, and functional characteristics in two groups of pregnant women: **(A)** the relative abundance of vaginal microbiota between the premature labor group and the term group was analyzed at the phylum and genus levels. **(B,C)** Linear discriminant analysis effect size (LEfSe) was performed to identify differentially expressed microbiota between the premature labor group and the term group. **(D)** PICRUSt2 analysis was used to identify differentially altered pathways based on differential vaginal microbiota.

The study analysis indicated that the composition of the vaginal microbiota had underwent changes at different levels. At the phylum level, the *Firmicutes*, *Proteobacteria*, *Actinobacteriota*, and *Bacteroidota* phyla exhibited higher relative abundances in pregnant women with sPTB and FTB, respectively. Among these, the *Firmicutes* phylum accounted for 76.73% of the vaginal microbiota in sPTB, while in FTB it accounted for 54.96%. The *Proteobacteria* phylum was the second most abundant phylum, accounting for 10.89% of the vaginal microbiota in sPTB and 25.21% in FTB. Additionally, *Lactobacillus, Pelomonas, Gardnerella, Ralstonia, Staphylococcus, Enterococcus, Prevotella, Ureaplasma, Escherichia-Shigella*, and *Bacteroides* were the primary bacterial genera at the genus level. Among these, *Lactobacillus* was the most abundant genus, accounting for 60.98% of the vaginal microbiota in sPTB and 43.07% in FTB. *Gardnerella* was the second most abundant genus, accounting for 7.21% of the vaginal microbiota in sPTB and 6.49% in FTB. In addition, *Staphylococcus, Enterococcus,* and *Ureaplasma* were also relatively abundant genera, accounting for 5.78, 3.64, and 2.44% of the vaginal microbiota in sPTB, and 1.3, 1.98, and 0.84% in FTB (Figure 2A).

Based on Linear Discriminant Analysis Effect Size (LEfSe) analysis with an LDA score > 2.0 and bubble charts, the differential bacterial species between the sPTB group and the FTB group can be visually displayed. Compared with pregnant women in the FTB group, the relative abundance of *Firmicutes* at the phylum level in pregnant women in the sPTB group was significantly increased (*p* < 0.05). However, the relative abundances of *Proteobacteria, Bacteroidota, Verrucomicrobiota, Patescibacteria, Cyanobacteria,* and *Spirochaetota* were significantly decreased (*p* < 0.05). Additionally, compared with the FTB group, the relative abundance of *Lactobacillus jensenii, Gardnerella vaginalis,* and *Deinococcus* sp. *Lab-1* was significantly increased in the sPTB group, while the relative abundance of *Brevundimonas vesicularis, Akkermansia muciniphila, Eubacterium rectale ATCC 33656, Bacteroides uniformis, Bacteroides caccae, Blautia obeum, Brevundimonas staleyi, Bacteroides thetaio, Eubacterium hallii, Collinsella aerofaciens, Roseburia inulinivorans, Intestinibacter bartlettii DSM 16795, Plesiomonas shigelloides, Alistipes indistinctus YIT 12060,* and *Dysgonomonas gadei* showed a significant decrease in relative abundance (Figures 2B,C). The above results indicate that the composition of microbiota flora in pregnant women with sPTB is significantly altered, which may be one of the key factors leading to premature labor.

To further identify the pathway changes associated with the differential vaginal microbiota in pregnant women with sPTB, PICRUSt2 analysis was used to identify the differentially altered pathways based on the differential vaginal microbiota by comparing with the KEGG database. Compared with FTB pregnant women, secondary bile acid biosynthesis and *Staphylococcus aureus* infection were significantly upregulated in sPTB pregnant women (*p* < 0.05). However, the synthesis and degradation of ketone bodies, biosynthesis of valine, leucine and isoleucine, biosynthesis of acid regurgitation and CoA, biosynthesis of ansamycin, metabolism of alanine, aspartate and glutamate, bacterial chemotaxis, biosynthesis of folate, metabolism of niacin and nicotinamide, biosynthesis of streptomycin, metabolism of C5-branched dibasic acids, degradation of fatty acids, biosynthesis of phenylalanine, tyrosine and tryptophan, sulfur metabolism, and biosynthesis of lipopolysaccharide were all significantly downregulated (*p* < 0.05) (Figure 2D).

### 3.4 Vaginal metabolomics analysis in preterm pregnant women

**Figure 3:**
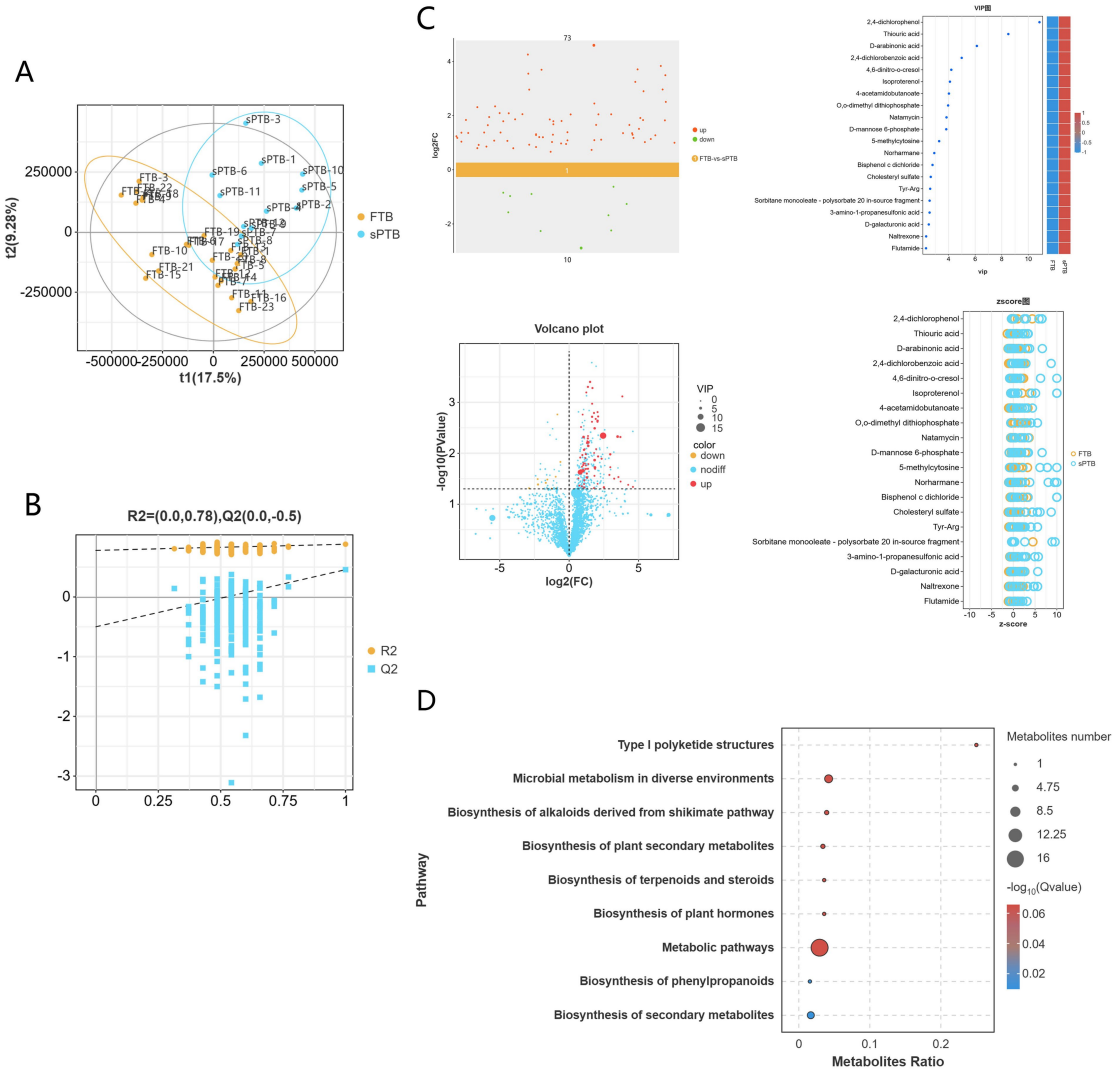
Metabolite analysis of vaginal samples from two groups of pregnant women: **(A)** an orthogonal partial least squares discriminant analysis (OPLS-DA) score plot was generated to visually demonstrate the differences in metabolic profiles between the FTP and sPTB groups. **(B)** A permutation plot of the OPLS-DA model was used to validate the robustness and reliability of the model. **(C)** Scatter plots, volcano plots, VIP plots, and z-score plots all showed the overall trend of differences in metabolite levels between the two groups. **(D)** A KEGG enrichment pathway bubble plot was generated to highlight the pathways with the most annotated differentially abundant metabolites.

In this study, 1,936 identified metabolites were identified in the positive ion mode (POS), 1,651 identified metabolites were identified in the negative ion mode (NEG), and a total of 3,587 metabolites were obtained in the mixed mode following combined analysis. These metabolites belong to different biochemical categories, including organic acids and their derivatives, lipids and lipoid molecules, organic cyclic compounds, benzene compounds, organic oxides, and phenylacetone and polyketone compounds, among others. We performed PLS-DA analysis on the above 3,587 metabolites. In the mixed positive and negative ion mode, there was significant heterogeneity between early pregnancy and full-term samples (Figure 3A); further PLS-DA rpermutation test (Figure 3B) showed that Q2Y < R2Y, indicating that the model had no overfitting phenomenon. Therefore, the model in this study showed good stability and could be used for subsequent analysis.

Based on the PLS-DA model, differential metabolites were screened with VIP > 1.0 and *p* < 0.05 as thresholds. A total of 83 differential metabolites were identified in the mixed mode, among which 73 metabolites were up-regulated and 10 metabolites were down-regulated in the premature labor group. The top 20 metabolites were plotted into VIP bubble charts and z-score charts. It can be seen that there are 5 kinds of Benzenoids: 2,4-dichlorophenol, 4,6-dinitro-o-cresol, Isoproterenol, Naltrexone, Flutamide; 4 kinds of organic oxides: D-arabinonic acid, Natamycin, D-mannose 6-phosphate, D-galacturonic acid; 4 kinds of organic acids and their derivatives: 2,4-dichlorobenzoic acid, 4-acetamidobutanoate, Tyr-Arg, 3-amino-1-propanesulfonic acid; 3 kinds of organic cyclic compounds: Thiouric acid, 5-methylcytosine, Norharmane; 1 kind of lipid and lipid-like molecule: Cholesteryl sulfate; 1 kind of organophosphorus compound: O,0-dimethyl dithiophosphate; 1 kind of bisphenol compound: bisphenol c dichloride; 1 kind of surfactant: sorbitan monooleate-polysorbate. The aforementioned 20 metabolites were significantly upregulated in pregnant in preterm pregnant women (Figure 3C).

Subsequently, KEGG pathway enrichment analysis was performed on the differential metabolites between the two groups of pregnant women. The results showed that the differential metabolites between the FTP group and the sPRT group were significantly enriched in 9 metabolic pathways (Figure 3D). Among them, the differential metabolites were significantly enriched in metabolic pathways such as type I polyketide structure, microbial metabolism, alkaloid synthesis via the shikimate pathway, biosynthesis of plant secondary metabolites, biosynthesis of terpenoids and steroids, biosynthesis of plant hormones, metabolic pathway, biosynthesis of phenylpropanoids, and biosynthesis of secondary metabolites.

### 3.5 Analysis of the mutual association among clinical inflammatory markers, vaginal microbiota, and metabolites in preterm pregnant women

**Figure 4:**
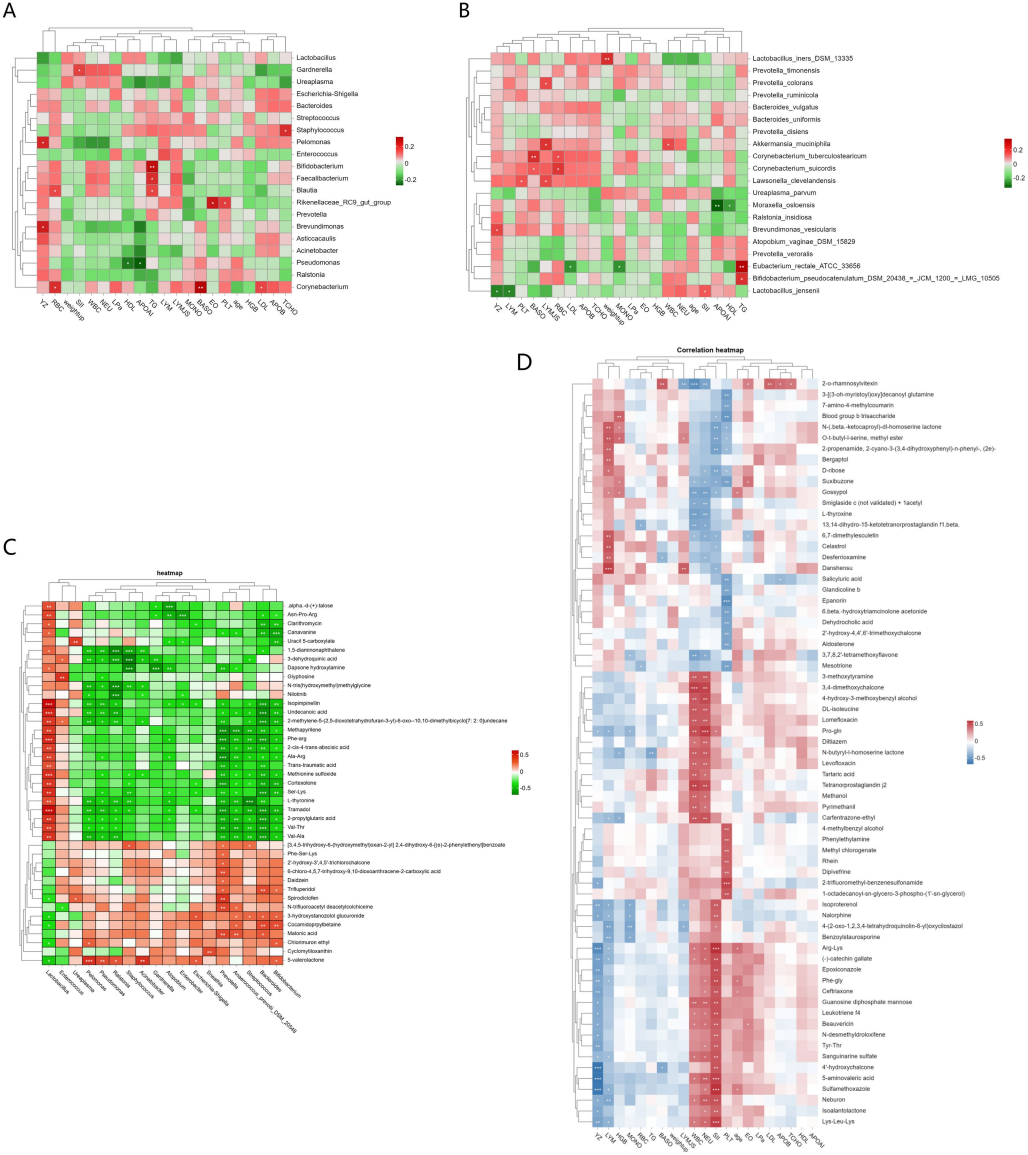
Analysis of mutual associations among clinical inflammatory markers, vaginal microbiota, and metabolites in pregnant women: **(A)** Heatmap of the correlation between inflammatory markers and vaginal microbiota (genus level) **(B)** Heatmap of correlation between inflammatory markers and vaginal microbiota (species level) **(C)** Heatmap of the correlation between vaginal microbiota and metabolites **(D)** Heatmap of the correlation between inflammatory markers, vaginal metabolites, and clinical factors (* indicates *p* < 0.05, ** indicates *p* < 0.01).

First, Pearson correlation analysis was performed between clinical inflammatory markers and vaginal microbiota to reveal the impact of inflammatory markers on microbial community structure. The study analysed the clinical data of pregnant women, such as YZ (gestational week), age, weight up (weight gain), WBC (white blood cell count), MONO% (monocyte percentage), LYM% (lymphocyte percentage), BASO% (basophil percentage), EO% (eosinophil percentage), NEU (neutrophil count), PLT (platelet count), RBC (red blood cell count), HGB (hemoglobin), LDL (low-density lipoprotein), TG (triglyceride), HDL (high-density lipoprotein), LPa (serum lipoprotein a), APOAI (apolipoprotein AI), APOB (apolipoprotein B), TCHO (total cholesterol), and SII (systemic immune-inflammation index), along with their vaginal microbiota. The results showed (Figure 4A) that at the genus level, *Pelomonas* and *Brevundimonas* were significantly positively correlated with gestational week (YZ) (*p* < 0.05), *Gardnerella* was significantly positively correlated with systemic immune-inflammation index (SII) (*p* < 0.05), *Bifidobacterium* was significantly positively correlated with triglyceride (TG) (*p* < 0.05), *Staphylococcus* was significantly positively correlated with total cholesterol (TCHO) (*p* < 0.05), and *Pseudomonas* was significantly negatively correlated with high-density lipoprotein (HDL) and apolipoprotein AI (AOLAI) (*p* < 0.05). Further analysis at the species level revealed (Figure 4B) that *Lactobacillus iners* was significantly positively correlated with weight up (*p* < 0.01), *Lactobacillus jensenii* was significantly negatively correlated with gestational week (YG) and lymphocyte (LYM) (*p* < 0.05), while being significantly positively correlated with systemic immune-inflammation index (SII) (*p* < 0.05), *Prevotella colorans*, *Akkermansia muciniphila,* and Lawsonella *clevelandensis* were significantly positively correlated with lymphocyte count (*p* < 0.05), amongst which *Lawsonella clevelandensis* was also significantly positively correlated with platelet (*p* < 0.05), and *Eubacterium rectale ATCC 33656* was significantly positively correlated with TG (triglyceride) (*p* < 0.01), and significantly negatively correlated with low-density lipoprotein (LDL) and MONO (monocyte) (*p* < 0.05).

Second, Pearson correlation analysis was performed between clinical inflammatory indicators and vaginal metabolites. The results showed (Figure 4D) that there were 4 categories of metabolites related to inflammatory factors (SII, WBC, NEU, LYM). The first category, mainly including metabolites such as arginine-lysine, sulfamethoxazole, lysine-leucine, 5-aminovaleric acid, phenylalanine-glycine, catechin gallate, epoxiconazole, leukotriene f4, and guanosine diphosphate mannose, was significantly positively correlated with the systemic immune-inflammation index (SII) (*p* < 0.05), and also positively correlated with white blood cells (WBC) and neutrophils (NEU). The second category of metabolites, such as 3,4-dimethoxychalcone, 2-O-rhamnosyl fucoside, prostaglandin j2, proline-glutamine, ethylmethprylon, 4-hydroxy-3-methoxybenzyl alcohol, DL-isoleucine, lomefloxacin, 3-methoxytyramine, N-butyrylhomoserine lactone, tartaric acid, azoxystrobin, methanol, levofloxacin, and diltiazem, showed a significant positive correlation with white blood cells (WBC) and neutrophils (NEU) (*p* < 0.05); both of these categories of metabolites were significantly negatively correlated with gestational age (YZ) (*p* < 0.05). The third category of metabolites mainly includes danshensu, selagibool, O-t-butyl-l-serine methyl ester, 6,7-dimethylraphanusin, N-(*β*-keto)-dl-homoserine lactone, 2-propenamide, 2-cyano-3-(3,4-dihydroxyphenyl)-n-phenyl, deferoxamine, ergothioneine and other metabolites, which are significantly positively correlated with lymphocytes (LYM) and negatively correlated with SII, WBC and NEU. The final category is metabolites that are positively correlated with gestational week (YZ) and significantly negatively correlated with SII, WBC and NEU: 2-propenamide, 2-cyano-3-(3,4-dihydroxyphenyl)-n-phenyl, (2e), N-(β-)-dl-homoserine lactone, O-t-butyl-l-serine methyl ester, D-ribose, 2-O-rhamnosylfucoside, L-thyroxine, 3,7,8,2′-tetramethoxyflavone, gexibool, 13,14-dihydro-15-ketotetrahydroprostaglandin.

Finally, a heatmap was generated using Spearman’s correlation to illustrate the correlation between vaginal microbiota and their metabolites. The results showed two groups of genera with correlation patterns with metabolites (Figure 4C). The first group of metabolites was significantly positively correlated with *Lactobacillus* (*p* < 0.05): *α*-d-(+)-talose, asparagine-proline-arginine, clarithromycin, canavanine, 5-carboxyuracil, 1,5-diaminonaphthalene, 3-epi-oleanolic acid, hydroxylamine dapsone, glyphosate, N-tris(hydroxymethyl)methylglycine, nilotinib, isopapaverine, undecanoic acid, 2-methylene-5-(2,5-dioxotetrahydrofuran-3-yl)-6-oxo-10,10-dimethylbicyclo[7:2:0]undecane, methylpyrrolidine, phenylalanine-arginine, 2-cis-4-trans-abscisic acid, alanine-arginine, trans-traumatic acid, sulfinimide, cortisol, serine-lysine, L-sarcosine, tramadol, 2-propylglutaric acid, valine-threonine, valine-alanine. This group of metabolites exhibited a negative correlation with the genera *Enterococcus*, *Ureaplasma*, *Pelomonas*, *Pseudomonas*, *Ralstonia*, *Staphylococcus*, *Acinetobacter*, *Gardnerella*, *Atopobium*, *Enterobacter*, *Escherichia-Shigella*, *Sneathia*, *Prevotella*, *Anaerococcus_prevotii_DSM_20548*, *Streptococcus*, *Bacteroides*, *Bifidobacterium* (*p* < 0.05); the other group was significantly negatively correlated with *Lactobacillus* (*p* < 0.05): benzoic acid, phenylalanine-serine-lysine, 2-hydroxy-3′,4,5′-trichlorochalcone, 6-chloro-4,5,7-trihydroxy-9,10-dioxoanthracene-2-carboxylic acid, daidzin, trifluperidol, pyridonecarboxylic acid ibuprofen, N-trifluoroacetyldehydroacetic colchicine, 3-hydroxystanozolol glucuronide, cocamidopropyl betaine, malonic acid, chlorpyriethyl, cyclostrombine, *δ*-valerolactone, and this group of metabolites was significantly positively correlated with *Enterococcus*, *Ureaplasma*, *Pelomonas*, etc. (*p* < 0.05).

## 4 Comment

Preterm birth (PTB) refers to delivery between 28 weeks and less than 37 weeks of gestation, and the newborn delivered at this time is termed a premature infant. There are approximately 15 million premature infants worldwide each year (Chawanpaiboon et al., 2019; Ohuma et al., 2023), with a global incidence rate of preterm birth ranging from 5 to 18% (Ansari et al., 2021), among which about 15% of premature infants die in the neonatal period. Preterm birth has become the second leading cause of neonatal death. Currently, clinical medical history and ultrasound examination play a crucial role in the prediction of preterm birth (Hoffman, 2021), but there is still a lack of reliable biomarkers, leading to insufficient prediction accuracy and suboptimal timing of intervention. Studies have shown that changes in the vaginal microbiome are closely related to the risk of preterm birth, and in particular, the imbalance of *Lactobacillus* may lead to adverse pregnancy outcomes (Fettweis et al., 2019; Stout et al., 2017; Kosti et al., 2020; Fudaba et al., 2021; Talukdar et al., 2024; Zhang et al., 2025b). The metabolites produced or modified by these types of microorganisms can have potential local and systemic effects on the host, contributing to the development of diseases (Thaiss et al., 2016; Yoshimoto et al., 2013; Witkowski et al., 2020; Buffa et al., 2022; Hao et al., 2024). This has driven the advancement of metabolomics, and researches on such microbiome-metabolome interactions have been delved into various human diseases, yielding potential mechanistic insights (Yachida et al., 2019; Lloyd-Price et al., 2019). Therefore, revisiting the field of the female reproductive system, the exploration of microbial and metabolic markers associated with preterm labor holds significant clinical relevance. It is anticipated that this study may contribute valuable insights for the early identification and timely intervention of preterm labor.

### 4.1 Principal findings

The vaginal microbiota plays a crucial role in maintaining pregnancy health. In this study, we investigated the dynamic changes in vaginal microbiota and metabolites in preterm pregnant women and their association with preterm birth risk. The study found that *α* diversity of vaginal microbiota was significantly reduced in the preterm group, which was closely associated with the occurrence of preterm birth. In terms of species composition, the relative abundance of *Lactobacillus, Gardnerella, Staphylococcus, Enterococcus,* and *Ureaplasma* were significantly increased in the preterm birth group, particularly *Lactobacillus jensenii.* These data indicated that microdysbiosis (especially the loss of balance of *Lactobacillus* flora and the increase of *Gardnerella*, etc.) led to a reduction in lactic acid, an increase in amine concentration, and the induction of inflammatory factor release, which may be important factors contributing to premature labor.

In terms of metabolites, 83 differential metabolites were found in the sPTB group, of which 73 were up-regulated and 10 were down-regulated. These up-regulated metabolites in the sPTB group, such as tyrosine-arginine, cholesterol sulfate, 2,4-dichlorophenol and other phenolic compounds, organic acids, and heterocyclic compounds, may influence the vaginal microenvironment. KEGG pathway analysis revealed 12 enriched pathways for differentially expressed metabolites, with the main enriched pathways being: Amino acid metabolism, Lipid biosynthesis, Microbial metabolism, Secondary metabolite biosynthesis, Terpenoid and steroid biosynthesis, Phytohormone biosynthesis, etc.

Additionally, this study analysed the correlations among the vaginal microbiota, metabolites, and clinical factors and found that inflammatory indicators were significantly elevated in the preterm group, such as SII was strongly correlated with Bacterial Vaginosis(BV) common vaginal *Gardnerella* spp. and its significant elevation in preterm pregnant women suggests that SII may be correlated with vaginal dysbiosis by reflecting systemic inflammation. Similarly, there is a correlation between some of the characteristic metabolites of the vagina and indicators of inflammation, such as arginine, lysine, sulfamethoxazole, 5-aminopentanoic acid, epoxiconazole, etc. Finally, this study also revealed new findings regarding *Lactobacillus*: *Lactobacillus iners* was positively correlated with weight gain, and *Lactobacillus jensenii* was negatively correlated with gestational weeks, that is, the higher the abundance, the shorter the gestational weeks and the higher the risk of premature labor, and there was a strong correlation between *Lactobacillus jensenii* and SII (a brief summary is provided in Table 2).

**Table 2:** Comparison of vaginal microbiome, metabolites, and inflammatory markers between sPTB and FTB groups.

Indicator category	Specific indicator	sPTB (*n* = 23)	FTB (*n* = 54)	*p*-value
Inflammatory markers	SII	1,061	689	*p* < 0.001
Vaginal microbiota diversity	*α* diversity (Shannon index)	2.65	3.56	*p* < 0.01
Phylum level	*Firmicutes*	76.73%	54.96%	*p* < 0.001
	*Proteobacteria*	10.89%	25.21%	*p* < 0.01
Genus level	*Lactobacillus*	60.98%	43.07%	*p* < 0.01
	*Gardnerella*	7.21%	6.49%	*p* = 0.15
	*Staphylococcus*	5.78%	1.30%	*p* < 0.01
	*Enterococcus*	3.64%	1.98%	*p* < 0.05
	*Ureaplasma*	2.44%	0.84%	*p* < 0.01
Specific bacterial strains	*L. jensenii*	Increase (dominant)	Lower	*p* < 0.05, negatively correlated with gestational age
Metabolic differences*	Number of significantly different metabolites (e.g., tyrosine-arginine, etc.)	73 types (primarily increases) 10 types (primarily decreases)	-	*p* < 0.05
KEGG pathways*	Activation pathways metabolism	Significant activation	Unactivated	*p* < 0.05

*Indicates sPTB (*N* = 12), FTB (*N* = 23).

### 4.2 Results in the context of what is known

Currently, there are more and more studies on *Lactobacillus* species. In the vagina of healthy reproductive-aged females, there is a community state dominated by *Lactobacillus*. *Lactobacillus* reduces the vaginal pH value by producing lactic acid to prevent the growth of pathogens (O'Hanlon et al., 2013), and meanwhile produces antibiotics, etc. Antibiotics can jointly protect the health of the female reproductive tract by maintaining membrane integrity (Delgado-Diaz et al., 2019). In 2011, Professor Ravel’s team classified the common species in the healthy vaginal flora into community state types (CSTs) (Ravel et al., 2011): CST-I (*Lactobacillus crispatus*), CST-II (*Lactobacillus gasseri*), CST-III (*Lactobacillus iners*), and CST-V (*Lactobacillus jensenii*); most of the above CSTs can produce significant amounts of lactic acid (Alonzo Martínez et al., 2021; Tsementzi et al., 2020). The remaining CST-IV represents a community with reduction of *Lactobacillus*, including *Atopobium*, *Gardnerella*, *Prevotella* and *Megasphaera*, and these colonies are closely related to bacterial vaginosis (BV) (Bradshaw et al., 2025). Numerous previous studies have shown that low abundance of CST-I (*Lactobacillus crispatus*) and high abundance of CST-IV (such as BV) are linked to an increased risk of premature labor (Fettweis et al., 2019; Stout et al., 2017; Kosti et al., 2020; Fudaba et al., 2021; Talukdar et al., 2024; Zhang et al., 2025b). As the main beneficial bacterium in the vaginal microbiome, *Lactobacillus crispatus* maintains the vaginal acidic environment and inhibits pathogen invasion by producing lactic acid, hydrogen peroxide and bacteriocins, which has been confirmed to be closely associated with vaginal health. In recent years, research progress primarily focused on its genomic diversity (Tarracchini et al., 2023), probiotic applications (Ravel et al., 2025), immunoregulatory mechanisms (Decout et al., 2024), and its role in preventing vaginal infections and adverse pregnancy outcomes (Wright et al., 2021). *Lactobacillus iners* is a common *Lactobacillus* species in the female vaginal microbiota, and its role in reproductive health has garnered attention in recent years. It can be detected in healthy vaginas and is also frequently associated with vaginal dysbiosis (Petrova et al., 2017; Holm et al., 2023; Bloom et al., 2022). Existing research indicates an association between *Lactobacillus iners* and obesity (particularly in the cervical microbiome) (Oh et al., 2015). Obesity may influence microbiome composition through metabolism, diet, or lifestyle, and in turn, the microbiota may further impact metabolic health (Kelly et al., 2024). In this study, we found that *Lactobacillus iners* was positively correlated with weight gain, suggesting its potential metabolic role and possible association with lipid metabolism during pregnancy. *Lactobacillus gasseri* (*L. gasseri*) is also an important protective bacterial species for female reproductive tract health. Most current studies support that *L. gasseri* maintains the balance of vaginal microbiota, inhibits the growth of pathogenic bacteria, and regulates immunisation function (Selle and Klaenhammer, 2013; Sawada et al., 2022). *Lactobacillus jensenii* represents another significant *lactobacillus* within the vaginal microbiome. Traditionally, it was believed to maintain the acidic vaginal environment through the production of lactic acid and hydrogen peroxide, thereby inhibiting pathogen growth. However, recent studies have revealed its complex role within the female reproductive tract. Strain-specific variations influence its protective or potentially pathogenic properties, particularly in the context of vaginal dysbiosis and adverse pregnancy outcomes such as preterm birth. *Lactobacillus jensenii* is another important *lactobacillus* in the vaginal microbiome, which is traditionally believed to maintain the vaginal acidic environment by producing lactic acid and hydrogen peroxide, thereby inhibiting the growth of pathogens. However, recent studies have revealed that its role in the female reproductive tract is complex, with strain-specific differences affecting its protective or potential pathogenic properties, especially in cases of vaginal dysbacteriosis and adverse pregnancy outcomes (such as premature labor) (Nori et al., 2023). A 2023 study published in Microbial Genomics, titled ‘*Profiling of vaginal Lactobacillus jensenii isolated from preterm and full-term pregnancies reveals strain-specific factors relating to host*’, found through genomic analysis that *L. jensenii* strains from preterm pregnant women were genetically distinct from those of full-term pregnant women. Multiple studies have found that the vaginal microbiota undergoes dynamic changes during various stages of pregnancy, and the pro-inflammatory effects of specific microorganisms may vary with the duration of pregnancy. For example, in early pregnancy (<14 weeks), the vaginal microbiota is relatively stable with high diversity, predominantly dominated by *Lactobacillus crispatus*, low abundance of *Lactobacillus jensenii*, and low risk of inflammation (Gupta et al., 2020). In the intermediate stage [second stage] of pregnancy (14–28 weeks): the abundance of *Lactobacillus* gradually increases (such as *L. crispatus* and *L. iners*), *α* diversity decreases, and the microbiota tends to be simplified to enhance the protective effect; however, if it shifts to non-*Lactobacillus* dominance, it may trigger inflammation (DiGiulio et al., 2015). In the advanced stage of pregnancy (28 weeks to delivery): the microbiota becomes more stable, with greater significant *Lactobacillus* dominance and the lowest diversity, which is conducive to maintaining pregnancy. At this time, the impact of increased abundance of *L. jensenii* on premature labor is weaker than that in the intermediate stage of pregnancy, because its inflammatory cascade may be triggered by other factors, such as Vaginitis bacterial or fungal infection, etc., however, it may still exacerbate the risk of Premature rupture of membranes through pro-inflammatory metabolites (Neumann et al., 2024). Studies have shown that during pregnancy, CST-V (*Lactobacillus jensenii*) synergizes with CST-III (*Lactobacillus iners*) as a microbial community in transition between CSTs (Jakobsson and Forsum, 2007; Verstraelen et al., 2009), and an increase in *Lactobacillus jensenii* may indicate unsteadiness of the microbial community (Verstraelen et al., 2009).

*Gardnerella* is commonly present in the vagina of women of childbearing age, and high relative abundance of *Gardnerella* may cause a series of female reproductive tract diseases. It is currently widely used to establish bacterial vaginosis models (Kwak et al., 2023), as it disrupts protective factors in the vagina by producing sialidase and proline aminopeptidase, and promotes the adhesion of other anaerobic bacteria (such as *Prevotella*) to the vaginal mucosal surface (Muzny et al., 2020). *Prevotella* releases amino acids to release amines, while *Gardnerella* utilises these amines to synthesise amino acids. In this process, the concentration of amines increases and the level of lactic acid decreases; their synergy can also promote the synthesis of lipopolysaccharides by symbiotic bacteria and induce the release of pro-inflammatory cytokines (Muzny et al., 2020), which further increases the risk of premature labor (Gao et al., 2021). *Ureaplasma* includes *Ureaplasma parvum* (Up) and *Ureaplasma urealyticum* (Uu), which can decompose urea through urease, increase vaginal pH, disrupt the microecological balance dominated by *lactobacilli*, induce inflammatory responses, and increase leukocyte and prostaglandin levels, thereby increasing the risk of infertility (Tamarelle et al., 2019). During pregnancy, it upregulates pro-inflammatory cytokines in amniotic fluid, leading to premature rupture of fetal membranes and premature birth (Sprong et al., 2020; Motomura et al., 2020). Furthermore, vaginal fungal infection, especially *Candida albicans*, is also closely associated with the occurrence of premature labor. *Candida albicans* has the ability to form biofilms, and the use of protective mucus enables *Candida albicans* to firmly adhere to the mucosal surface and remain in a closed environment that many systemic antifungal agents cannot penetrate, thereby maintaining the infection, especially in patients with decreased immunity (such as pregnant women) (Bradshaw et al., 2025). Multiple studies have shown that vaginal *C. albicans* colonization or infection during pregnancy can increase the risk of premature labor (Messina et al., 2024; Eskezia et al., 2025), and mother-to-fetus transmission can lead to invasive neonatal infection or adverse fetal outcomes (Farr et al., 2015). Additionally, *Enterococcus* and *Staphylococcus* have also been found in recent years to be associated with spontaneous abortion, preterm birth (Ardissone et al., 2014), and neonatal infectious diseases (Deng et al., 2019). Although this is not entirely consistent with previous study results, this study also found that the increase in *Lactobacillus jensenii* is closely associated with the risk of preterm birth, which aligns with *L. jensenii*’s recent studies, suggesting that changes in *lactobacilli* species are associated with the risk of preterm birth. We did not identify fungal infection in the samples of the preterm labor group in this study, which may be because most of the preterm labor samples had fetal membranes rupture before admission, and the flushing of amniotic fluid cleaned the vagina, thus no fungi were detected. Furthermore, *β*-diversity analysis of the microbiota revealed significant compositional differences between the FTP and sPTB groups, further supporting the potential link between preterm birth risk and microbiota changes.

Metabolites produced or modified by microorganisms have been found to cause disease with potential local and systemic effects on the host (Thaiss et al., 2016). The major metabolic compounds up-regulated in this study may not only disrupt the vaginal microbiome but also activate the immune system, leading to inflammation: cholesterol sulfate is a lipid that stabilises cell membranes and regulates signalling pathways such as protein kinase C and PI3K; if disrupted, it may exacerbate inflammation (Kindschuh et al., 2023). Tyrosine-arginine, an amino acid derivative, may regulate immune responses through the urea pathway, leading to inflammation-related cascades associated with PTB (Pruski et al., 2021; Proietti et al., 2020). Aromatic compounds (e.g., 2,4-dichlorophenol) and organic acids (e.g., 2,4-dichlorobenzoic acid) may be produced by microbial metabolism, altering vaginal pH and promoting PTB-associated pathogens such as *Gardnerella vaginalis* (Zhang et al., 2025a). Organic heterocyclic compounds (e.g., 5-methylcytosine, norman) may reflect microbial or host metabolic activity, influencing the vaginal ecosystem (Liu et al., 2021). These metabolic products have potential as biomarkers for early PTB risk detection or therapeutic targets for regulating inflammation and microbial balance (Kosti et al., 2020), but their causal roles remain under investigation. The upregulation of these benzene compounds and inflammatory metabolites may be associated with inflammatory responses, suggesting that preterm birth may be closely related to local or systemic inflammatory states, consistent with existing research findings. In summary, these findings emphasise the importance of vaginal microbiota and metabolites in the mechanisms of preterm birth and provide potential biomarkers for future clinical interventions.

KEGG pathway reveals differential metabolite enrichment pathway. Amino acid metabolism: Tyrosine—Arginine is associated with phenylalanine and tyrosine metabolism and may influence immune regulation via kynurenine pathway (Pruski et al., 2021; Proietti et al., 2020). Lipid biosynthesis: Cholesterol sulfate is involved in steroid and lipid metabolism, affecting membrane stability and inflammatory signal transduction (Kindschuh et al., 2023). Microbial metabolism: Benzene compounds and organic acids (e.g., 2,4-dichlorophenol, 2,4-dichlorobenzoic acid) are associated with microbial activity, potentially driven by probiotics such as *Gardnerella* or *Prevotella*, altering the vaginal microenvironment (Zhang et al., 2025a). Secondary metabolite biosynthesis: The biosynthesis of phenylalanine, taxadienic acid, and polyketides produces compounds such as 2,4-dichlorobenzoic acid, which are related to microbial activity and inflammation (Liu et al., 2021). Terpenoid and steroid biosynthesis: These pathways contribute to lipid-related metabolic products and affect vaginal epithelial function (Kindschuh et al., 2023). Phytohormone biosynthesis: organic oxidised compounds (e.g., D-galacturonic acid) may reflect metabolic changes in microorganisms or the host (Zhang et al., 2025a). These pathways interact with the vaginal microbiota, and dysbiosis in the vaginal microbiota drive metabolic changes, leading to inflammatory changes and further resulting in premature birth (Fettweis et al., 2019).

Female reproductive tract health involves the function of organs such as the uterus, ovaries, and fallopian tubes. Inflammation may affect ovarian function, embryo quality, and implantation process, leading to adverse outcomes such as infertility and pregnancy loss (Chen et al., 2024; Zhang Z. et al., 2025; Li et al., 2023). The systemic immune inflammation index (SII) is an inflammatory marker calculated as platelet count (/L) × neutrophil count (/L) / lymphocyte count (/L), and has been widely used in recent years to assess the prognosis of inflammation-related diseases. In PCOS patients, women with higher SII have lower embryo utilisation rates and pregnancy rates in *in vitro* fertilisation (IVF) (Li et al., 2023). This association between SII and pregnancy loss suggests that it may serve as a predictive indicator of reproductive health risks. Currently, there is a lack of direct research on the relationship between SII and vaginal microbiota, but indirect studies have shown that components of SII (such as NLR) are elevated in BV patients (Pek et al., 2021). This study also found that SII is closely related to *Gardnerella vaginalis*, a common bacterium in BV, and its significant elevation in preterm pregnant women suggests that SII may reflect systemic inflammation and vaginal microbiota dysbiosis. Future studies should further validate this relationship to clarify the potential application of SII in vaginal health management. In addition, there is also some association between characteristic vaginal metabolites and inflammatory markers. Arginine is a semi-essential amino, which serves as a precursor to nitric oxide (NO) and generates NO through NO synthase (NOS), affecting vasodilation, platelet aggregation, and leukocyte function. NO plays an important role in immune regulatio, can enhance the activity of macrophages and T cells (Costa et al., 2016), and is involved in the body’s immune regulation and inflammatory responses. Studies have shown that oral arginine can significantly inhibit platelet aggregation in patients with hypercholesterolemia, and the mechanism is related to increased NO and elevated levels of cyclic guanosine monophosphate (cGMP) in platelets (Wolf et al., 1997). Lysine, an essential amino acid, competes with arginine for metabolic pathways and may influence inflammation by inhibiting viral replication or regulating white blood cell function. Studies have shown that lysine can induce inflammatory and immune responses (Gunarathne et al., 2025). These may indirectly affect the platelet count components of SII. Additionally, in mammals, lysine can enhance non-specific immunity in fish and regulate inflammation-related signalling pathways (such as p38 MAPK). Lysine may indirectly influence inflammation by inhibiting viral replication but is not directly associated with WBC, NEU, or LYM (Huang et al., 2021). In addition, this study found that metabolites such as sulfamethoxazole [a derivative of sulfanilamide, which may originate from environmental exposure or microbial metabolism (Chasekwa et al., 2025)], 5-aminovaleric acid [an intermediate in amino acid metabolism (Murakami et al., 2023; Cai et al., 2025)], and epoxiconazole [a benzene compound/agrochemical residue (Reiss et al., 2022)] are closely associated with SII. However, direct evidence for their causal association is still lacking in existing studies. By constructing an association network of microbiota-metabolites-inflammatory indicators and performing KEGG pathway enrichment analysis, we inferred their potential inflammatory relevance. Through enrichment analysis, we verified the embedding of these metabolites in inflammation-related pathways (such as the kynurenine pathway and microbial metabolic pathways), which are often associated with NF-κB activation and inflammation amplification. This suggests that they may act as microbially induced inflammatory mediators, promoting amino acid/lipid disturbances and systemic inflammation (increased SII), even though their exact mechanism is unknown.

### 4.3 Research implications

*Firmicutes*, especially the genus *Lactobacillus*, maintain an acidic environment and barrier function under healthy conditions (Chee et al., 2020). Under pathological conditions (such as BV, vaginal dysbiosis, HPV infection, or premature labor), these bacterial species interconvert through CST conversion: CST I (dominated by *L. crispatus*, the most stable) can shift to CST III (dominated by *L. iners*, transitional type) or CST IV (high diversity, non-*Lactobacillus* dominated); CST III (*L. iners*) is the most unstable and easily shifts to CST IV under hormonal changes, antibiotics, or infection, leading to inflammation amplification and pathogen invasion; CST II (*L. gasseri*) and CST V (*L. jensenii*) are relatively protective but can shift to CST III or IV under increased pH or inflammatory stress, involving mechanisms such as reductions in lactic acid production, metabolite disturbances, and activation of immune signals (Buchta et al., 2025; Dong et al., 2024). This transformation reflects the dynamics of the microbiome, shifting from protective species to an unstable or pathogenic state, thereby increasing the risk of infection. In this study, the increased abundance of *Firmicutes* in the premature labor group also indicates that specific *Firmicutes* (such as *Lactobacillus*) may dominate but do not necessarily confer a protective effect (Huang et al., 2023). The increased abundance of *Lactobacillus jensenii* in the premature labor group was negatively correlated with gestational weeks (i.e., the higher the abundance, the shorter the gestational weeks and the greater the risk of premature labor), suggesting that it may play a detrimental role in vaginal microbiome dysregulation, leading to amplified inflammation during pregnancy, reduced lactic acid levels, and increased pH, thereby promoting premature labor cascade reactions such as uterine contractions and premature rupture of membranes. This clinically emphasizes that atypical *Lactobacillus* species (such as those dominated by *L. jensenii*) may not be protective but rather risk factors associated with specific strains (Nori et al., 2023), especially in white or Asian pregnant populations (Cavanagh et al., 2025). This finding supports enhanced monitoring of the vaginal microbiome during the second trimester of pregnancy, such as assessing the abundance of *L. jensenii* through 16S rRNA sequencing, to identify high-risk pregnant women (Cavanagh et al., 2025). This negative correlation suggests that the abundance of *L. jensenii* could serve as a potential early biomarker, especially when combined with its genomic traits (such as unique clades and metabolic gene variants of term strains), for predicting the risk of premature labor (Nori et al., 2023). However, its reliability needs to be verified through larger-scale prospective studies. Current evidence indicates that its predominance in the second trimester can be part of the risk indicators for sPTB (spontaneous preterm birth) rather than an independent marker (Nori et al., 2023; Cavanagh et al., 2025). In addition, this study shows a significant difference in the relative abundance of *Lactobacillus jensenii* between the premature labor group and the term group, suggesting that certain strains may lack protective functions (such as maintaining the vaginal acidic environment or inhibiting inflammation). Compared with the full-term group, specific genetic differences in the strains of the premature labor group include unique phylogenetic clades, which may also involve differences in genome variants or metabolic pathways; thereby weakening the immune interaction with the host, reducing lactic acid production and enhancing the potential for inflammation induction, leading to microbial dysbiosis and amplifying the risk of premature labor. These findings highlight the importance of strain-level analysis and support its use as a potential biomarker, but further validation is required.

Overall, this study focuses on the association between the increased inflammatory index SII and vaginal microbiota imbalance (increased abundances of *Lactobacillus jensenii*, *Gardnerella*, *Staphylococcu*s, *Enterococcu*s, *Ureaplasma*, etc.), metabolite changes (including upregulated amino acids such as tyrosine-arginine and arginine-lysine), and significant activation of the kynurenine pathway in KEGG pathway analysis in pregnant women with premature labor. These findings suggest that the inflammatory mechanism of premature labor may originate from a cascade reaction induced by microbe-metabolite interactions. Previous studies have shown that LPS-induced inflammation can increase the expression of kynurenine-forming enzymes (IDO and TDO) and kynurenine in the placenta through NF-κB activation, leading to elevated inflammatory factors (such as TNF-*α* and IL-6). This process is common in premature labor models, and NF-κB inhibitors (such as sulfasalazine) can reverse this activation, suggesting that NF-κB may be an upstream signalling pathway of kynurenine metabolism. Additionally, animal models of premature labor also support that infection/inflammation triggers NF-κB-mediated bidirectional maternal-fetal inflammation. Moreover, existing literature indicates that bacterial infection can activate the cGAS-STING pathway by releasing DNA or metabolites, and cGAS-STING is an important component of the human innate immune response, playing a key role in infection, cell stress, and tissue injury. Therefore, this study hypothesizes that the specific activated inflammatory pathway may be: microbial infection (such as vaginal microbiota dysbiosis) enhances tryptophan metabolism, activates the NF-κB signalling pathway, promotes inflammation, and ultimately leads to the occurrence of premature labor. This hypothesis emphasizes the role of NF-κB as a core inflammatory pathway under kynurenine activation, which may drive premature labor through microbially induced metabolic changes. Of course, it is assumed that further experiments are needed to verify the causal relationship. Therefore, future studies can conduct *in vitro* and *in vivo* experiments based on this hypothesis, such as constructing in vitro cell models or premature labor mouse models, to verify how tryptophan metabolism induces inflammation through the NF-κB signalling pathway via microbiota intervention/IDO inhibitor, and explore its mechanism of action in premature labor. In addition, a premature labor risk prediction model will be constructed by machine learning to provide a new theoretical basis and targeted strategies for high-risk prediction, target intervention and precise treatment of premature labor.

### 4.4 Strengths and limitations

This study integrates multi-omics approaches, including 16S rRNA sequencing, untargeted metabolomics, and inflammatory profiling, to comprehensively investigate the interplay between vaginal microbiota, metabolic alterations, and inflammatory responses in preterm birth. The combined analysis of microbial composition, metabolite pathways, and systemic inflammatory indices provides a more holistic understanding of the mechanisms underlying inflammation-induced preterm labor. The inclusion of both microbiome and host inflammatory data enhances the translational value and potential clinical relevance of the findings.

However, there are still several limitations. First, the sample size is relatively small, which may affect the statistical power and the generalizability of the findings. In this study, the sample size of the premature labor group was only *n* = 23 (total sample *n* = 77), which is relatively small, especially in observational studies involving multivariate analyses (such as 16S RNA sequencing, metabolomics, and correlation analysis), which may weaken the reliability of the results. In terms of statistical power, a small sample size typically results in low statistical power, meaning that the study’s ability to detect true effects is insufficient. Specifically, 1. Increased risk of type I and type II errors: when a true difference exists, a small sample may not achieve sufficient power, thus missing significance (false negative); 2. Multiple comparison problem: the study involves multiple variables (such as 83 differential metabolites and KEGG pathways), and a small sample is prone to type I errors (false positive) or may miss true correlations due to insufficient power, for example, the association between *Lactobacillus jensenii* and inflammation may be falsified in a larger sample. In terms of generality, small sample size limits the roll out of results to a larger population. 1. Insufficient representativeness: The samples of this study were from Shanghai, which may lead to selection bias. For example, the changes in the flora of the premature labor group (such as the increase of *Lactobacillus jensenii*) may not reflect the diversity of pregnant women globally, impacting external validity; 2. High variability: Microbiological and metabolomics data are highly individualized, and small samples are difficult to capture population variants, leading to conclusions (such as activation of the kynurenine pathway) that may be overfitted to specific groups and cannot be generalized to different ethnic groups or other stages of pregnancy. Secondly, there is currently a lack of research on the inflammatory pathways that may be activated by microbe-induced metabolite changes driving premature labor, as well as further in vivo and in vitro experiments to verify the causal relationship. Currently, research on bacteria is in full swing, including interactions between bacteria and bacteria and between bacteria and hosts, has also proposed the latest ‘quorum sensing (QS)’ method. As quorum sensing communication occurs among microbiota, when quorum sensing signal molecules (QSSM) released by bacteria are perceived by the host, relevant signalling pathways are activated, which activates or inhibits the expression of related genes, resulting in a series of metabolites and cytokines (Zeng et al., 2023). Such small molecule metabolites in the human body may affect host physiology and, at the same time, inversely continue to influence the communication state of these microbial communities, thereby increasing the risk of adverse reproductive health. Thereby increasing the risk of adverse reproductive health outcomes (Yachida et al., 2019; Yang et al., 2023). Therefore, exploring the interaction mechanisms between microbial communities and metabolites can aid in understanding their causal relationships. Additionally, the study did not track the long-term effects on postpartum mother-infant outcomes, limiting a comprehensive assessment of the potential consequences of preterm birth.

Future research can be optimized through the following aspects: 1. Expand the sample size and enhance statistical power through multi-center collaboration (such as joint collaboration among multiple hospitals or research institutions), which can reduce the occurrence of type II errors. 2. Optimize the study design and conduct prospective design studies: that is, recruit participants from early pregnancy, dynamically monitor changes in vaginal flora and metabolites, avoid recall bias in retrospective studies, and improve causal inference ability. 3. Targeted recruitment: focus on high-risk premature labor populations (such as those with a history of vaginal infection), increase the proportion of the case group, and optimize the efficiency of sample utilization. Finally, supplement animal model verification and construct animal models of premature labor: reproduce the mechanism by which vaginal flora imbalance induces premature labor in mouse models, verify the causal relationship of the hypothesized kynurenine-NF-κB pathway, make up for the limitations of small human samples, and promote clinical application in the future.

## 5 Conclusion

Vaginal dysbacteriosis, particularly the increased abundance of *Lactobacillus jensenii*, may be activated through pathways such as kynurenine and lipid metabolism, resulting in increased production of pro-inflammatory metabolites, thereby activating inflammation to drive premature labor. In the future, clinically, the risk may be reduced through screening and targeted interventions (such as probiotics or IDO inhibition) in the middle and advanced stages of pregnancy. Further studies should verify the causal mechanism and optimize intervention strategies.

## Data Availability

The raw sequence reads analyzed in the study are deposited in the NCBI repository, accession number PRJNA1330029. The non-targeted metabolomics data are deposited in the OMIX, China National Center for Bioinformation/Beijing Institute of Genomics, Chinese Academy of Sciences (https://ngdc.cncb.ac.cn/omix), accession number OMIX012026.
